# *ALKBH1* Gene rs6494 T>A Polymorphism Decreases Wilms Tumour Risk in Chinese Children

**DOI:** 10.1111/jcmm.70864

**Published:** 2025-09-28

**Authors:** Changmi Deng, Haixia Zhou, Na Zhang, Min Chen, Rui‐Xi Hua, Jiwen Cheng, Suhong Li, Jiao Zhang, Jichen Ruan, Wen Fu, Jing He, Guochang Liu

**Affiliations:** ^1^ Department of Pediatric Surgery, Guangzhou Institute of Pediatrics, Guangdong Provincial Key Laboratory of Research in Structural Birth Defect Disease, Guangzhou Women and Children's Medical Center Guangzhou Medical University, Guangdong Provincial Clinical Research Center for Child Health Guangzhou Guangdong China; ^2^ Department of Hematology, The Key Laboratory of Pediatric Hematology and Oncology Diseases of Wenzhou The Second Affiliated Hospital and Yuying Children's Hospital of Wenzhou Medical University Wenzhou Zhejiang China; ^3^ Department of Pathology The Affiliated Children's Hospital of Xi'an Jiaotong University Xi'an Shaanxi China; ^4^ Department of Pediatric Surgery The Second Affiliated Hospital of Xi'an Jiaotong University Xi'an Shaanxi China; ^5^ Department of Pathology Children Hospital and Women Health Center of Shanxi Taiyuan Shannxi China; ^6^ Department of Pediatric Surgery The First Affiliated Hospital of Zhengzhou University Zhengzhou Henan China

**Keywords:** *ALKBH1*, polymorphism, susceptibility, Wilms tumour

## Abstract

Wilms tumour (WT) is the most predominant renal carcinoma that affects children, and the understanding of the genetic mechanisms underlying WT development is continually evolving. The role of the demethylase ALKBH1, which is known for its association with diverse cancers, in WT has never been explored. Here, we aimed to investigate the associations between genetic variants of *ALKBH1* and WT risk in Chinese children. A total of 414 WT patients and 1199 healthy controls were recruited from five centres in China. Three polymorphisms (rs1048147, rs6494 and rs176942) of the *ALKBH1* gene were genotyped via the TaqMan genotyping assay. We found that rs6494 T>A was significantly associated with a reduced risk of WT [TA vs. TT: adjusted odds ratio (AOR) = 0.59, 95% confidence interval (CI) = 0.39–0.87, *p* = 0.009; TA/AA vs. TT: AOR = 0.61, 95% CI = 0.42–0.91, *p* = 0.014]. Stratification analysis revealed that the protective genotype of rs6494 (TA/AA) was significantly associated with reduced WT risk in subgroups with ages younger than 18 months, male sex and clinical stages III and III‐IV. Moreover, through eQTL analysis, we observed that rs6494 T>A was associated with reduced *ALKBH1* expression and elevated *SNW1* and *ADCK1* expression. We identified the rs6494 T>A polymorphism of the *ALKBH1* gene as a WT susceptibility locus, providing valuable insights into the etiology underlying WT susceptibility.

**Abbreviations**
ALKBH1: AlkB homologue 1
CI: confidence interval
eQTL: expression quantitative trait loci
GTEx: Genotype‐Tissue Expression
HWE: Hardy–Weinberg equilibrium
LD: linkage disequilibrium
lncRNA: long noncoding RNA
m^1^A: N^1^‐methyladenosine
MAF: minor allele frequency
OR: odds ratio
SNP: single nucleotide polymorphism
UTR: untranslated region
WT: Wilms tumour

## 1 Introduction

Wilms tumour (WT, nephroblastoma) is a childhood embryonal tumour intersecting disrupted organogenesis and tumorigenesis [1]. It accounts for 90% of childhood renal tumours and constitutes 7% of all childhood cancers [2]. Wilms tumour generally affects approximately 1 in 10,000 children [3], and its incidence differs across distinct ethnic populations [4]. The annual incidence of Wilms tumour in China is lower than that in North America or Europe (3.3 cases per million vs. 8.9 cases per million) [4, 5, 6]. Wilms tumour may occur sporadically or in the context of bilateral tumours, multifocal disease and specified genetic predisposition syndromes that frequently include either genitourinary malformation or overgrowth [3, 7]. In up to 15% of patients, Wilms tumour develops in the context of a predisposition syndrome or germline mutation in cancer risk‐associated genes [3]. This disease typically occurs before 7 years of age, with the mean age at diagnosis being 3.7 years in unilateral cases and 2.6 years in bilateral cases [7]. The occurrence of genetic predisposition syndromes and the earlier onset of bilateral tumours indicate the pivotal role of hereditary predisposition in Wilms tumour development [3]. The cancer‐associated genes that underlie Wilms tumour development are diverse and involve up to 40 genes, such as germline alterations in the *WT1* gene and epigenetic alterations in the 11p15 locus [3, 8, 9]. Despite this, much work remains to be done to identify additional risk factors that are responsible for the heritability of Wilms tumour susceptibility.

The molecular drivers of Wilms tumour development frequently include the blockade of genetic pathways that guide the normal embryogenesis of the genitourinary tract [10]. Abnormal epigenetic regulation, particularly aberrant nuclear methylation, plays pivotal roles in the initiation and progression of Wilms tumours by altering gene expression and function [10, 11, 12, 13]. N1‐methyladenosine (m1A) modification, which is an isomer of m6A that carries a methyl group at the N1 position, has been identified as a reversible modification of tRNA, mRNA, rRNA and long noncoding RNA (lncRNA) [14, 15, 16]. The methyl group in m1A, which has a positive electrostatic charge under physiological conditions, disrupts Watson‐Crick base pairing with uridine and consequently affects the secondary/tertiary structure of mRNAs as well as RNA–protein interactions [17]. m1A is regulated by methyltransferases (writers), such as TRMT6, TRMT61A, TRMT61B, TRMT10C and NML; demethylases (erasers), such as ALKBH1, ALKBH3, ALKBH7 and FTO; and m1A‐binding proteins (readers), such as YTHDF1, YTHDF2, YTHDF3 and YTHDC1 [18, 19]. The significance of m1A modification and its regulatory components in the tumorigenesis of various cancers, including glioma, oral squamous cell carcinoma, lung cancer, hepatocellular carcinoma, gastrointestinal cancer, colorectal cancer, ovarian cancer, breast cancer, urothelial carcinomas and prostate cancer, has attracted substantial attention [18]. Recently, investigations on Wilms tumour have highlighted the importance of m1A modification regulators. Notably, certain single nucleotide polymorphisms (SNPs) that are present in m1A writers (TRMT6), erasers (FTO), and readers (YTHDC1 and YTHDF2) have been associated with the risk of Wilms tumour risk [20, 21, 22, 23], emphasising the significant role of m1A modification regulators in Wilms tumour development.

The *ALKBH1* gene, which is also known as AlkB homologue 1 and histone H2A dioxygenase, belongs to a 2‐oxoglutarate (2OG)‐dependent dioxygenase family/group that consists of several well‐known DNA/RNA demethylases, such as TET, FTO and ALKBH1‐7 [24]. ALKBH1 demethylates m1A at the 58th position in tRNA, playing critical roles in regulating translation elongation [25, 26]. Previous studies have established a robust relationship between ALKBH1‐demethylated m1A modification and various cancers, such as pancreatic cancer and colorectal cancer [27, 28]. Concurrently, reports have emerged delineating the associations of SNPs in *ALKBH1* with the risk of developing gastric cancer and acute lymphoblastic [29, 30]. This accumulated evidence has led to the hypothesis that there is a potential association between ALKBH1 and the risk of Wilms tumour development. However, no studies have explored the potential impact of polymorphisms in *ALKBH1* on Wilms tumour risk thus far.

Here, we conducted a case–control study involving five centres and including 414 Wilms tumour patients and 1199 controls from the Chinese Han population. We aimed to explore the association between SNPs in the *ALKBH1* gene and susceptibility to Wilms tumour development.

## 2 Materials and Methods

### 2.1 Study Design and Participants

Detailed information about the subjects was previously reported [31, 32]. In brief, 414 patients with histopathologically confirmed Wilms tumour and 1199 matched control subjects were recruited (Table S1). These participants were enrolled from five hospitals in five different cities in China. All the controls were matched to the patients in terms of age and sex, had no history of Wilms tumour, and visited the same medical centre during the same period. The study followed the Declaration of Helsinki and was authorised by the Ethics Committee of Guangzhou Women and Children Medical Center (No. 202016601). Written consent was obtained from the participants' parents or legal guardians.

### 2.2 Polymorphism Selection and Genotyping

The dbSNP database, SNPinfo web database, and literature studies were utilised to identify candidate SNPs of the *ALKBH1* gene for the survey [33, 34, 35]. Sites of polymorphism were specifically selected on the basis of the following criteria: SNPs were located in the 5′‐untranslated region (5′‐UTR), 3′‐UTR, introns, or exons; SNPs had potential biological functions (affecting transcription factor‐binding sites, microRNA‐binding sites, or shear sites or leading to changes in coding amino acids); nonsynonymous SNPs were preferentially selected; minor allele frequencies (MAFs) were > 5% in the Chinese population; and there was no linkage disequilibrium (LD) between selected SNPs (*R* ^2^ < 0.8). Accordingly, *ALKBH1* rs1048147, rs6494 and rs176942 were selected for further study, as no significant LD was found between the selected SNPs (*R* ^2^ = 0.015 between rs6494 and rs1048147, *R* ^2^ = 0.008 between rs6494 and rs176942, *R* ^2^ = 0.095 between rs1048147 and rs176942).

Genomic DNA was extracted from samples using a TIANamp Blood DNA Kit (TianGen Biotech, Beijing, China). Gene polymorphisms were genotyped using TaqMan probe real‐time polymerase chain reaction (Applied Biosystems, Foster City, CA) [33]. A 10% replicate sample, negative control and positive control were included in each 384‐well plate to verify the reliability of the typing results.

### 2.3 Statistical Analysis

The genotypes of the SNPs were assessed for adherence to Hardy–Weinberg equilibrium (HWE) within the control sample via a goodness‐of‐fit chi‐square test. Differences in the distributions of demographic and clinical variables between the patients and controls were assessed using a two‐sided chi‐square test. To estimate the relative risk associated with each genotype, odds ratios (ORs) with corresponding 95% confidence intervals (CIs) and two‐sided *P* values were calculated using unconditional logistic regression. Additionally, associations were evaluated by stratifying the groups on the basis of age, sex and clinical stage. Moreover, expression quantitative trait loci (eQTL) analysis was conducted via the Genotype‐Tissue Expression (GTEx) project (https://www.gtexportal.org/) [36] to investigate the associations between the genotypes of candidate SNPs and the mRNA expression levels of genes. Statistical significance was defined as a P value less than 0.05. All the statistical analyses were performed using SAS version 9.1 software (SAS Institute Inc., Cary, NC).

## 3 Results

### 3.1 Effect of *ALKBH1* Gene SNPs on Wilms Tumour Risk

The clinical characteristics of the participants were described in our previous study (Table S1) [32]. Three SNPs in the *ALKBH1* gene (rs1048147 C>A, rs6494 T>A and rs176942 A>G) were successfully genotyped in a total of 398 patients and 1198 controls. As shown in Table 1, all the SNPs in the controls were in Hardy–Weinberg equilibrium (HWE, *p* > 0.05). The associations between these SNPs and Wilms tumour risk were calculated by unconditional logistic regression adjusted for sex and age. Among these SNPs, rs6494 was markedly associated with a reduced risk of Wilms tumour development (TA vs. TT: adjusted OR = 0.59, 95% CI = 0.39–0.87, *p* = 0.009; additive: adjusted OR = 0.66, 95% CI = 0.45–0.96, *p* = 0.028; dominant: adjusted OR = 0.61, 95% CI = 0.42–0.91, *p* = 0.014). In contrast, although not significant, a tendency toward a protective effect was observed for rs1048147 C>A and rs176942 A>G. We then defined rs1048147 CA/AA, rs6494 TA/AA and rs176942 AG/GG as protective genotypes on the basis of their ORs. Individuals with 1–3 protective genotypes presented a 0.71‐fold decrease in the risk of developing Wilms tumour compared with those with 0 protective genotypes (95% CI = 0.55–0.92, *p* = 0.009). Additionally, we conducted haplotype analysis (Table 2) and found that haplotype A‐A‐A, which spans SNPs rs1048147, rs6494 and rs176942, was significantly associated with a reduced risk of Wilms tumour development (OR = 0.43, 95% CI = 0.20–0.91, *p* = 0.027), suggesting a potential protective effect of the haplotype against Wilms tumour.

**TABLE 1:** Associations between *ALKBH1* gene polymorphisms and Wilms tumour risk.

Genotype	Cases (*N* = 398)	Controls (*N* = 1198)	*p* ^a^	Crude OR (95% CI)	*p*	Adjusted OR (95% CI)^b^	*p* ^b^
rs1048147 C>A (HWE = 0.949)							
CC	213 (53.52)	600 (50.08)		1.00		1.00	
CA	158 (39.70)	495 (41.32)		0.90 (0.71–1.14)	0.381	0.90 (0.71–1.14)	0.380
AA	27 (6.78)	103 (8.60)		0.74 (0.47–1.16)	0.188	0.73 (0.47–1.15)	0.180
Additive			0.156	0.88 (0.73–1.05)	0.156	0.88 (0.73–1.05)	0.151
Dominant	185 (46.48)	598 (49.92)	0.235	0.87 (0.69–1.09)	0.235	0.87 (0.69–1.09)	0.232
Recessive	371 (93.22)	1095 (91.40)	0.252	0.77 (0.50–1.20)	0.253	0.77 (0.50–1.20)	0.243
rs6494 T>A (HWE = 0.116)							
TT	364 (91.46)	1039 (86.73)		1.00		1.00	
TA	32 (8.04)	157 (13.11)		**0.58 (0.39–0.87)**	**0.008**	**0.59 (0.39–0.87)**	**0.009**
AA	2 (0.50)	2 (0.17)		2.85 (0.40–20.34)	0.295	2.91 (0.41–20.73)	0.287
Additive			0.024	**0.65 (0.45–0.95)**	**0.025**	**0.66 (0.45–0.96)**	**0.028**
Dominant	34 (8.54)	159 (13.27)	0.012	**0.61 (0.41–0.90)**	**0.013**	**0.61 (0.42–0.91)**	**0.014**
Recessive	396 (99.50)	1196 (99.83)	0.246	3.01 (0.42–21.46)	0.271	3.06 (0.43–21.81)	0.265
rs176942 A>G (HWE = 0.636)							
AA	274 (68.84)	809 (67.53)		1.00		1.00	
AG	112 (28.14)	348 (29.05)		0.95 (0.74–1.22)	0.693	0.95 (0.74–1.23)	0.709
GG	12 (3.02)	41 (3.42)		0.86 (0.45–1.67)	0.664	0.86 (0.45–1.67)	0.659
Additive			0.584	0.94 (0.76–1.16)	0.584	0.94 (0.77–1.17)	0.593
Dominant	124 (31.16)	389 (32.47)	0.627	0.94 (0.74–1.20)	0.627	0.94 (0.74–1.20)	0.639
Recessive	386 (96.98)	1157 (96.58)	0.694	0.88 (0.46–1.69)	0.695	0.88 (0.46–1.68)	0.689
Combined effect of protective genotypes^c^							
0	115 (28.89)	268 (22.37)		1.00		1.00	
1–3	283 (71.11)	930 (77.63)	0.008	**0.71 (0.55–0.92)**	**0.008**	**0.71 (0.55–0.92)**	**0.009**

*Note:* The values were in bold is the *p* value less than 0.05 or the 95% CI excluding 1.00.

Abbreviations: CI, confidence interval, HWE, Hardy–Weinberg equilibrium; OR, odds ratio.

^a^ *χ* ^2^ test for genotype distributions between Wilms tumour patients and cancer‐free controls.

^b^ Adjusted for age and sex.

^c^ Protective genotypes were carriers with rs1048147 CA/AA, rs6494 TA/AA and rs176942 AG/GG genotypes.

**TABLE 2:** The frequency of inferred haplotypes of *ALKBH1* gene based on observed genotypes and their association with Wilms tumour risk.

Haplotypes^a^	Cases (*n* = 796)	Controls (*n* = 2396)	Crude OR (95% CI)	*p*	Adjusted OR^b^ (95% CI)	*p* ^b^
CTA	472 (59.30)	1320 (55.09)	1.00		1.00	
CTG	85 (10.68)	273 (11.39)	0.87 (0.67–1.14)	0.306	0.87 (0.67–1.14)	0.317
CAA	22 (2.76)	71 (2.96)	0.87 (0.53–1.41)	0.566	0.87 (0.53–1.42)	0.572
CAG	5 (0.63)	31 (1.29)	0.45 (0.17–1.17)	0.101	0.45 (0.18–1.17)	0.103
ATA	158 (19.85)	522 (21.79)	0.85 (0.69–1.04)	0.114	0.85 (0.69–1.04)	0.111
ATG	45 (5.65)	120 (5.01)	1.05 (0.73–1.51)	0.795	1.05 (0.73–1.50)	0.811
AAA	8 (1.01)	53 (2.21)	**0.42 (0.20–0.89)**	**0.024**	**0.43 (0.20–0.91)**	**0.027**
AAG	1 (0.13)	6 (0.25)	0.47 (0.06–3.88)	0.480	0.47 (0.06–3.93)	0.487

*Note:* The values were in bold is the *p* value less than 0.05 or the 95% CI excluding 1.00.

Abbreviations: CI, confidence interval; OR, odds ratio.

^a^ The haplotypes order was rs1048147, rs6494 and rs176942.

^b^ Obtained in logistic regression models with adjustment for age and gender.

### 3.2 Stratification Analysis

We then analysed the associations between the *ALKBH1* gene polymorphisms and susceptibility to Wilms tumour development in subgroups separated by age, sex and clinical stage (Table 3). Stratification analysis revealed that the protective genotype TA/AA of rs6494 was significantly associated with a reduced risk of Wilms tumour development in subgroups with age younger than 18 months (adjusted OR = 0.45, 95% CI = 0.22–0.93, *p* = 0.031), male sex (adjusted OR = 0.57, 95% CI = 0.35–0.95, *p* = 0.031), clinical stage III disease (adjusted OR = 0.23, 95% CI = 0.07–0.73, *p* = 0.013) and stage III‐IV disease (adjusted OR = 0.26, 95% CI = 0.10–0.65, *p* = 0.004). Additionally, individuals with 1–3 protective genotypes also presented a reduced risk of Wilms tumour development in subgroups with male sex (adjusted OR = 0.67, 95% CI = 0.48–0.95, *p* = 0.026) and clinical stage II (adjusted OR = 0.64, 95% CI = 0.42–0.98, *p* = 0.038).

**TABLE 3:** Stratification analysis for association between *ALKBH1* genotypes and Wilms tumour risk.

Variables	rs6494 (case/control)		AOR (95% CI)^a^	*p* ^a^	Protective genotypes (case/control)		AOR (95% CI)^a^	*p* ^a^
	TT	TA/AA			0	1–3		
Age, month								
≤ 18	127/403	9/62	**0.45 (0.22–0.93)**	**0.031**	43/110	93/355	0.67 (0.44–1.02)	0.063
> 18	237/636	25/97	0.71 (0.45–1.14)	0.155	72/158	190/575	0.73 (0.53–1.01)	0.061
Sex								
Female	171/465	14/56	0.68 (0.37–1.25)	0.216	53/122	132/399	0.76 (0.52–1.11)	0.157
Male	193/574	20/103	**0.57 (0.35–0.95)**	**0.031**	62/146	151/531	**0.67 (0.48–0.95)**	**0.026**
Clinical stage								
I	119/1039	18/159	1.00 (0.59–1.69)	0.997	34/268	103/930	0.89 (0.59–1.34)	0.564
II	102/1039	10/159	0.65 (0.33–1.26)	0.201	35/268	77/930	**0.64 (0.42–0.98)**	**0.038**
III	87/1039	3/159	**0.23 (0.07–0.73)**	**0.013**	23/268	67/930	0.83 (0.51–1.37)	0.470
IV	41/1039	2/159	0.33 (0.08–1.37)	0.127	15/268	28/930	0.54 (0.28–1.02)	0.057
I + II	221/1039	28/159	0.84 (0.54–1.28)	0.412	69/268	180/930	0.76 (0.56–1.04)	0.085
III + IV	128/1039	5/159	**0.26 (0.10–0.65)**	**0.004**	38/268	95/930	0.72 (0.48–1.07)	0.102

*Note:* The values were in bold is the *p* value less than 0.05 or the 95% CI excluding 1.00.

Abbreviations: AOR, adjusted odds ratio; CI, confidence interval.

^a^ Adjusted for age and sex, without the stratify factor.

### 3.3 Effect of rs6494 on Gene Expression

**FIGURE 1:**
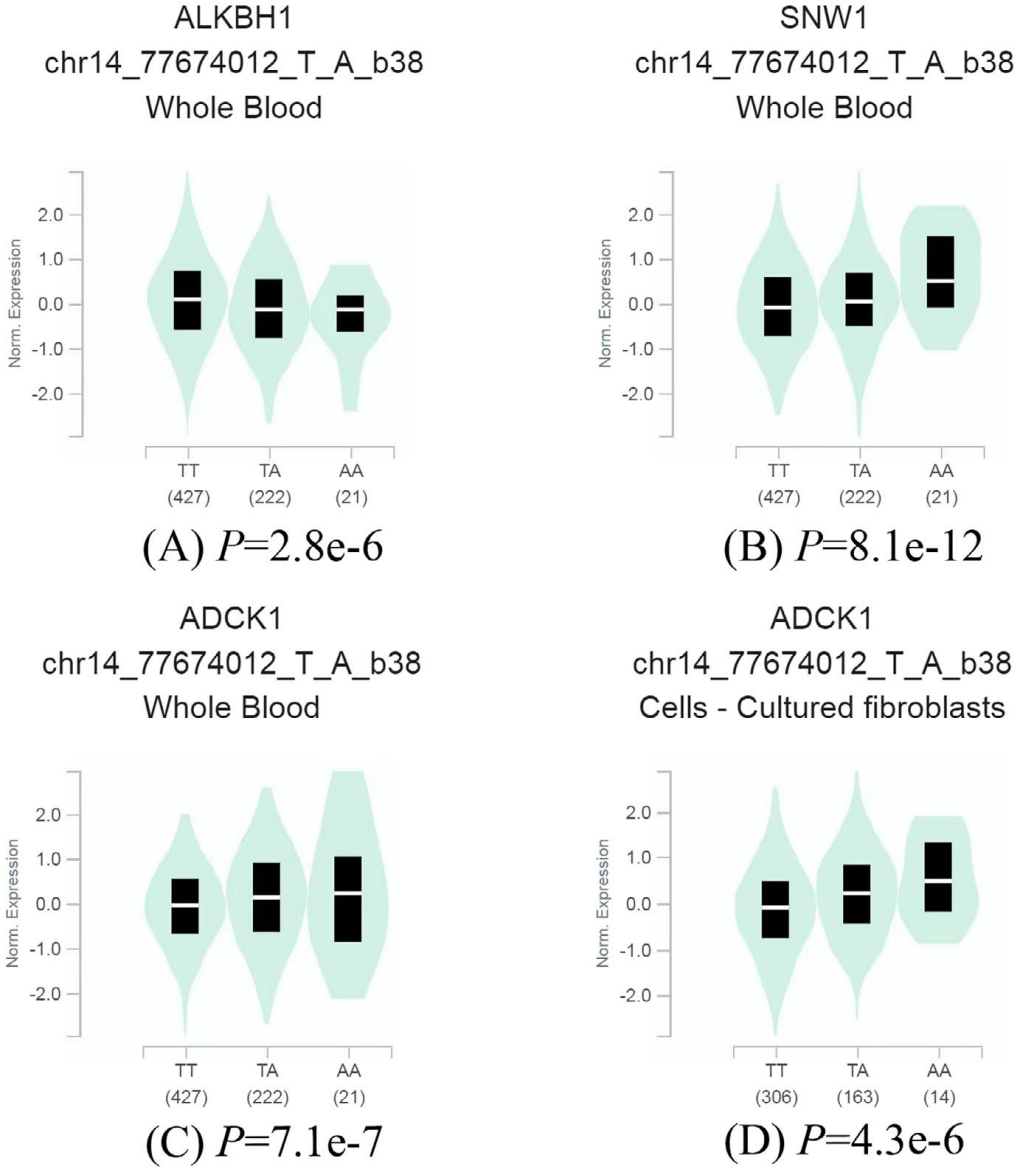
Functional relevance of rs6494 on gene expression in the GTEx database. The rs6494 was significantly associated with the mRNA expression levels of (A) *ALKBH1* (*p* = 2.8 × 10^−6^), (B) *SNW1* (*p* = 8.1 × 10^−12^) and (C) *ADCK1* (*p* = 7.1 × 10^−7^) in whole blood, as well as the mRNA expression level of (D) *ADCK1* (*p* = 4.3 × 10^−6^) in cell‐cultured fibroblasts.

We subsequently utilised the GTEx database to investigate the relationship between the rs6494 T>A polymorphism and changes in gene expression. Compared with the risk allele T of rs6494, the protective allele A of rs6494 was associated with decreased mRNA expression of *ALKBH1* (*p* = 2.8 × 10^−6^) and increased mRNA expression of *SNW1* (*p* = 8.1 × 10^−12^) and *ADCK1* (*p* = 7.1 × 10^−7^) in whole blood (Figure 1A–C). Additionally, in cultured fibroblasts, the protective allele A of rs6494 was associated with increased mRNA expression of *ADCK1* (*p* = 4.3 × 10^−6^) compared with the risk allele T of rs6494 (Figure 1D).

## 4 Discussion

This is the first multicenter epidemiological study to investigate the associations between *ALKBH1* genetic polymorphisms and risk of developing Wilms tumour in Chinese children. Our results revealed a significant association between the rs6494 polymorphism and susceptibility to Wilms tumour development, and the protective allele A of rs6494 was associated with decreased expression of *ALKBH1* and increased expression of *SNW1* and *ADCK1*. These observations highlight the potential biological implications of genetic polymorphisms of *ALKBH1* in the pathogenesis of Wilms tumour.

In this study, we investigated the potential roles of three SNPs (rs6494, rs1048147 and rs176942) in the *ALKBH1* gene and their associations with susceptibility to Wilms tumour development. When these SNPs were analysed individually, only rs6494 was significantly associated with the risk of Wilms tumour development. Located in the 3'UTR of *ALKBH1*, rs6494 T>A may affect microRNA binding, leading to the downregulation of *ALKBH1* and the concurrent upregulation of *SNW1* and *ADCK1*, which are two genes that have been implicated in transcriptional regulation and mitochondrial function, respectively [37, 38]. This altered expression pattern suggests that rs6494 may contribute to tumorigenesis through the dysregulation of DNA repair, cell cycle and metabolic pathways. Although rs1048147 and rs176942, which are located in intronic and splicing‐adjacent regions, respectively, did not exhibit significant associations with the risk of Wilms tumour development when assessed independently, combined genotype analysis and haplotype‐based analysis involving all three SNPs revealed statistically significant correlations with disease susceptibility. These findings suggest a potential synergistic or cumulative effect of these variants in modulating ALKBH1 function or expression, highlighting the importance of SNP interactions in the genetic predisposition to Wilms tumour development. Further mechanistic studies are warranted to elucidate the functional relevance of these SNPs in the pathogenesis of Wilms tumour.

In this study, we observed an association between the protective allele A of rs6494 and the reduced expression of the *ALKBH1* gene. The rs6494 is located in the coding region of the *ALKBH1* gene and results in a mutation from the T allele to the A allele, resulting in an amino acid substitution from methionine (Met, M) to leucine (Leu, L). A family‐based susceptibility study in Hispanic patients, which focused on childhood B‐cell acute lymphoblastic leukaemia, revealed that rs6494 may be a maternal SNP that potentially influences phenotype development in offspring [30]. Furthermore, research on *ALKBH1*−/− knockout mice has revealed the significant role of ALKBH1 in placental trophoblast lineage differentiation [39]. Overall, the impact of rs6494 on *ALKBH1* potentially involves the regulation of nephrogenesis lineage differentiation, which contributes to the development of Wilms tumour.

ALKBH1 participates not only in the m1A modification of tRNA but also in the m6A modification of DNA, along with various other posttranscriptional methylation modifications of RNAs, including m3C, m5C and m6A [40, 41, 42, 43, 44]. This raises an interesting question about which ALKBH1‐mediated methylation modifications contribute to the development of Wilms tumour. It is reasonable to hypothesize that ALKBH1 most likely participates in the development of Wilms tumour by regulating the m1A modification of RNA, given the numerous SNPs that are associated with Wilms tumour risk and found in m1A modification genes [20, 21, 22, 23]. However, accumulating studies indicate that ALKBH1 contributes to the development of a range of cancers through diverse modification patterns, and its role across different cancer types is inconsistent. For example, in pancreatic cancer, ALKBH1 functions as a demethylase of m1A modification, participating in the occurrence and development of pancreatic cancer through the mTOR and ErbB signalling pathways, and its low expression is related to the poor prognosis of patients [27]. Conversely, ALKBH1 is overexpressed in lung cancer, gastric cancer, glioblastoma and colorectal cancer and its overexpression markedly enhances viability and migration [28, 45, 46, 47]. Despite similar biological phenotypes, the pattern of ALKBH1 modification and its molecular process varies among these cancers. For example, in colorectal cancer, ALKBH1‐mediated m1A demethylation of METTL3 mRNA promotes metastasis by downregulating SMAD7 expression [28]. In lung cancer, ALKBH1 regulates the m6A modification of mRNA, promoting lung cancer cell invasion and migration [45]. In glioblastoma, ALKBH1 dynamically regulates the m6A level of DNA, and its depletion leads to transcriptional silencing of oncogenic pathways by decreasing chromatin accessibility [47]. Taken together, these findings suggest that ALKBH1 may be involved in the development of Wilms tumour through diverse modification patterns. Further exploration of its potential biological mechanism is warranted.

In contrast to the effect of rs6494 on *ALKBH1* expression, we observed that the protective allele A of rs6494 is related to elevated expression of the *SNW1* and *ADCK1* genes. The *SNW1* gene, which is located downstream of the *ALKBH1* gene with a genomic distance of 10 kb, encodes a coactivator that enhances transcription from some Pol II promoters. This gene is highly conserved in splicing and transcription. Prior studies have reported its association with various diseases, including cancers such as breast cancer, neuroblastoma, retinoblastoma and noncancer diseases such as rheumatoid arthritis and diabetic kidney disease [48, 49, 50, 51, 52, 53]. The gene was identified as a critical regulator of spatial bone morphogenetic protein (BMP) activity in vertebrate embryos [37]. Given the pivotal roles of BMP signalling and its modifiers in multiple stages of kidney development [54, 55], it is plausible that the effect of rs6494 on *SNW1* may affect the BMP signalling pathway and potentially contribute to the pathogenesis of Wilms tumour. The *ADCK1* gene, which is similar to the *SNW1* gene, is also located downstream of the *ALKBH1* gene. This gene encodes the mitochondrial protein AarF domain‐containing kinase 1. Research has revealed that *ADCK1* gene overexpression in colon cancer and osteosarcoma contributes to tumour cell growth [56, 57]. However, the biological function of ADCK1 remains unknown. Consequently, to elucidate the role of rs6494 in the pathogenesis of Wilms tumour and to gain insights into its underlying biological mechanism, additional investigations to reveal the biological functions of SNW1 and ADCK1, as well as their potential involvement in the pathogenesis of Wilms tumour, are warranted.

## 5 Limitations of This Study

Several limitations of this study are worth discussing. First, the current sample size was relatively limited, and all the subjects in this study were of Han Chinese descent, hindering the power of the stratification analysis and the generalisation of the findings to non‐Han Chinese populations. Further validation cohorts involving diverse ethnic populations and larger sample sizes are warranted. Second, Wilms tumour is a complex disease that is influenced by numerous genetic factors, and only three SNPs in the *ALKBH1* gene cannot fully explain the risk of Wilms tumour development. A polygenetic risk assessment, considering the impact of all identified genetic variants, is imperative to comprehensively understand Wilms tumour susceptibility. Finally, the underlying mechanism by which SNPs in *ALKBH1* affect Wilms tumour risk awaits further investigation.

## 6 Conclusion

In conclusion, our study identified rs6494 T>A in the *ALKBH1* gene as a susceptibility locus for WT, providing valuable insights into the etiological factors underlying Wilms tumour susceptibility.

## Author Contributions

**Changmi Deng:** formal analysis (equal), investigation (equal), writing – original draft (equal). **Haixia Zhou:** funding acquisition (equal), investigation (equal), resources (equal), writing – original draft (equal). **Na Zhang:** investigation (equal), writing – original draft (equal). **Min Chen:** investigation (equal), resources (equal), writing – review and editing (equal). **Rui‐Xi Hua:** formal analysis (equal), funding acquisition (equal), investigation (equal), writing – review and editing (equal). **Jiwen Cheng:** investigation (equal), resources (equal), writing – review and editing (equal). **Suhong Li:** investigation (equal), resources (equal), writing – review and editing (equal). **Jiao Zhang:** investigation (equal), resources (equal), writing – review and editing (equal). **Jichen Ruan:** investigation (equal), resources (equal), writing – review and editing (equal). **Wen Fu:** investigation (equal), resources (equal), writing – review and editing (equal). **Jing He:** conceptualization (equal), formal analysis (equal), funding acquisition (equal), investigation (equal), resources (equal), supervision (equal), writing – review and editing (equal). **Guochang Liu:** investigation (equal), resources (equal), supervision (equal), writing – review and editing (equal).

## Ethics Statement

The study protocol was assessed and approved by the institutional review board of Guangzhou Women and Children's Medical Center (Ethical Approval No: 202016601).

## Consent

In accordance with the guidelines of the Declaration of Helsinki, each participant provided written informed consent.

## Conflicts of Interest

The authors declare no conflicts of interest.

## Supporting information


**Table S1:** Frequency distribution of selected variables in Wilms tumour patients and cancer‐free controls.

## Data Availability

All the data are available upon request.
